# Effects of a novel, 3D printed bilateral arytenoid abductor on canine laryngeal airway resistance ex vivo

**DOI:** 10.1186/s12917-022-03263-y

**Published:** 2022-05-20

**Authors:** Katelyn E. MacGillivray, Sean D. Bellefeuille, Daniel E. Hoffmann, Lindsay L. St. Germaine

**Affiliations:** 1 Veterinary Specialists and Emergency Services, Rochester, NY 14623 USA; 2 Maitland, Florida USA; 3 Department of Biomedical Engineering, Rochester Institute of Technology, Rochester, NY 14623 USA

**Keywords:** Laryngeal paralysis, Laryngeal airway resistance, Arytenoid abductor

## Abstract

**Background:**

Laryngeal paralysis is a disease process most commonly seen in older, large breed dogs. When both arytenoid cartilages are affected dogs can develop life-threatening respiratory compromise, therefore surgical intervention is recommended. While there are multiple surgical procedures that have been described to treat laryngeal paralysis, there remains a considerable risk for postoperative complications, most commonly aspiration pneumonia. The objective of this ex vivo experimental study was to evaluate the effects of a novel, 3D printed bilateral arytenoid abductor on laryngeal airway resistance in canine cadaver larynges. Laryngeal airway resistance was calculated for each specimen before (control) and after placement of a 3D printed, bilateral arytenoid abductor. The airway resistance was measured at an airflow of 10 L/min with the epiglottis closed and at airflows ranging from 15 L/min to 60 L/min with the epiglottis open. The effects of the bilateral arytenoid abductor on laryngeal airway resistance were evaluated statistically.

**Results:**

With the epiglottis open, median laryngeal airway resistance in all larynges with a bilateral arytenoid abductor were significantly decreased at airflows of 15 L/min (0.0cmH2O/L/sec), 30 L/min (0.2cmH2O/L/sec), and 45 L/min (0.2cmH2O/L/sec) compared to the controls 15 L/min (0.4cmH2O/L/sec; *P* = 0.04), 30 L/min (0.9cmH2O/L/sec; *P* = 0.04), and 45 L/min (1.2cmH2O/L/sec; *P* = 0.04). When the epiglottis was closed, there was no significant difference in laryngeal resistance between the control (18.8cmH_2_O/L/sec) and the abducted larynges (18.1cmH_2_O/L/sec; *P* = 0.83).

**Conclusions:**

Placement of a bilateral arytenoid abductor reduced laryngeal resistance in canine cadaver larynges compared to the controls when the epiglottis was open. With the epiglottis closed, there was no loss of laryngeal resistance while the device abducted the arytenoid cartilages. The results of this ex vivo study is encouraging for consideration of further evaluation of the bilateral arytenoid abductor to determine an appropriate material and tolerance of this device in vivo.

## Background

Laryngeal paralysis is a condition where the muscles that control the movement of the arytenoid cartilages of the larynx lose their innervation [1]. This leads to lack of abduction of the arytenoid cartilages during inspiration and subsequent increase in laryngeal airway resistance. Acquired laryngeal paralysis is thought to most commonly represent an early clinical sign of a generalized idiopathic polyneuropathy [2, 3], but it can also be associated with trauma, chronic endocrinopathies, neoplasia, infection, or iatrogenic injury [3–5]. When both arytenoid cartilages are affected dogs can present on an emergency basis for upper airway obstruction. Due to the risk for development of life-threatening respiratory compromise, surgical management is recommended. The ideal goal of surgical intervention is to decrease laryngeal airway resistance without increasing risk of aspiration pneumonia [6]. Previously described surgical procedures include unilateral or bilateral arytenoid lateralization, ventriculocordectomy, partial arytenoidectomy and castellated laryngofissure [4, 7–10]. Unilateral arytenoid lateralization is considered the gold standard surgical technique because of its consistent outcome and low postoperative morbidity and mortality rates [11–13]. However, complications of this procedure can be serious and potentially life-threatening, including recurrent airway obstruction due to surgical failure and postoperative aspiration pneumonia which may affect between 5–24% of postoperative patients [12–17]. In a normal dog, aspiration is prevented by apposition of the arytenoid cartilages, epiglottis and vocal cords, completely covering the rima glottidis. The unilateral arytenoid lateralization procedure abducts one arytenoid cartilage laterally increasing the diameter of the rima glottidis beyond that which can be effectively covered by the epiglottis, which then increases the risk of aspiration pneumonia [4, 13, 18]. Previous studies have suggested that symmetrically abducting both arytenoids to a lesser magnitude could decrease airway resistance while also protecting against aspiration pneumonia [4, 19]. A novel, 3D printed bilateral arytenoid abductor was developed to achieve this symmetrical arytenoid abduction which would decrease airway resistance in patients with laryngeal paralysis and allow for continued rima glottidis coverage postoperatively to prevent aspiration pneumonia.

The purpose of this study was to evaluate the effects of a novel, 3D printed bilateral arytenoid abductor on the laryngeal airway resistance of canine cadaveric larynges with the epiglottis open and closed. Our primary hypothesis was that the bilateral arytenoid abductor would lower calculated laryngeal airway resistance compared to the control with the epiglottis open. Our secondary hypothesis was that there would be no difference in laryngeal airway resistance with the bilateral arytenoid abductor in place compared to the control with the epiglottis closed.

## Methods

### Specimens

Laryngeal specimens were obtained from cadavers of 6 large breed dogs that were euthanized with EUTHASOL® Euthanasia Solution (pentobarbital sodium and phenytoin sodium) at the local shelter for reasons other than upper airway disease. All larynges appeared anatomically normal at the time of specimen retrieval. All extrinsic soft tissues were removed from each specimen leaving only the laryngeal cartilages, intrinsic laryngeal musculature, and the first 5 tracheal rings. Each larynx was wrapped in sponges moistened with saline (0.9% NaCl) solution and stored at 2 °C until testing which occurred within 1 week of collection. Laryngeal specimens were warmed at room temperature for ~ 12 h prior to testing.

### Measurement of airway resistance

A stay suture (3–0 polydioxanone) was inserted through the tip of the epiglottis and both suture ends were passed down the trachea and secured with a mosquito hemostat to create a handle allowing for adjustments to the position of epiglottis. Larynges were periodically moistened with saline solution throughout testing.

**Fig. 1:**
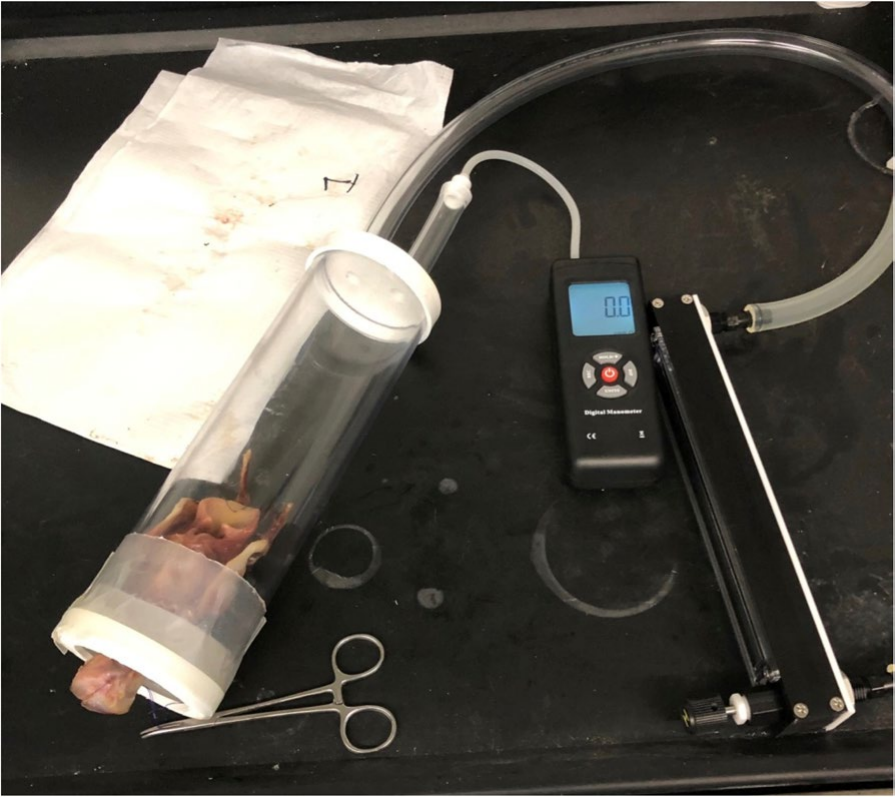
Image of a cadaver larynx mounted in the testing chamber for measurement of airway pressure. The inflow at the end of the chamber is connected to a high-flow air circuit. The outflow end has an airtight seal around the tracheal rings but the lumen of the trachea is continuous with the environment. The two ends of the stay suture for epiglottic manipulation have been passed down the trachea and attached to mosquito hemostats

For each experiment, the larynx was secured in a PVC chamber with an airtight seal around the tracheal rings, which were open to the atmosphere, similar to previously reported studies (Fig. 1) [4, 6]. At the opposite end of the chamber, a high-flow air circuit was attached. The airflow was determined by a pre-calibrated flowmeter (Riteflow aluminum flowmeter, Bel-Art Products, Pequannock, NJ) connected to a central high-pressure line. The pressure in the chamber was also measured at this end with a handheld digital manometer (Digi-Sense pressure and flowmeter, Cole-Parmer, Vernon Hills, IL). The system was tested for leaks by manual occlusion of the trachea resulting in a sustained elevation of pressure within the chamber. Airflow was set at a steady state for values between 15 and 60 L/min in increments of 15 L/min when the epiglottis was open, similar to previous reports [4]. The airway pressure for the control was first measured for each larynx with the epiglottis open. The epiglottis was then closed by pulling on the stay suture and airflow was reduced to 10 L/min and the airway pressure was measured. Pressure readings needed to remain at a constant value for 10 s prior to recording data. Each test was performed three times to ensure repeatability, such that the pressure difference varied by no more than 1 cmH_2_0. The three measured values were then recorded to the 100^th^ decimal place. The laryngeal airway resistance was then calculated as previously reported, [4, 18] using the following equation: LAR = ΔP/V, where P is the pressure gradient across the larynx and V is the airflow.

### Data analysis

The residuals were not normally distributed (normal probability plot and histogram) therefore, the median of the three replications was calculated. Comparisons of pre-post at each flow rate was by means of the Wilcoxon signed rank test. Data were reported as median and 25th /75th quartiles given the nonnormality of the residuals (percentiles); *P* < 0.05 was considered significant.

### Arytenoid abductor and placement technique

**Fig. 2:**
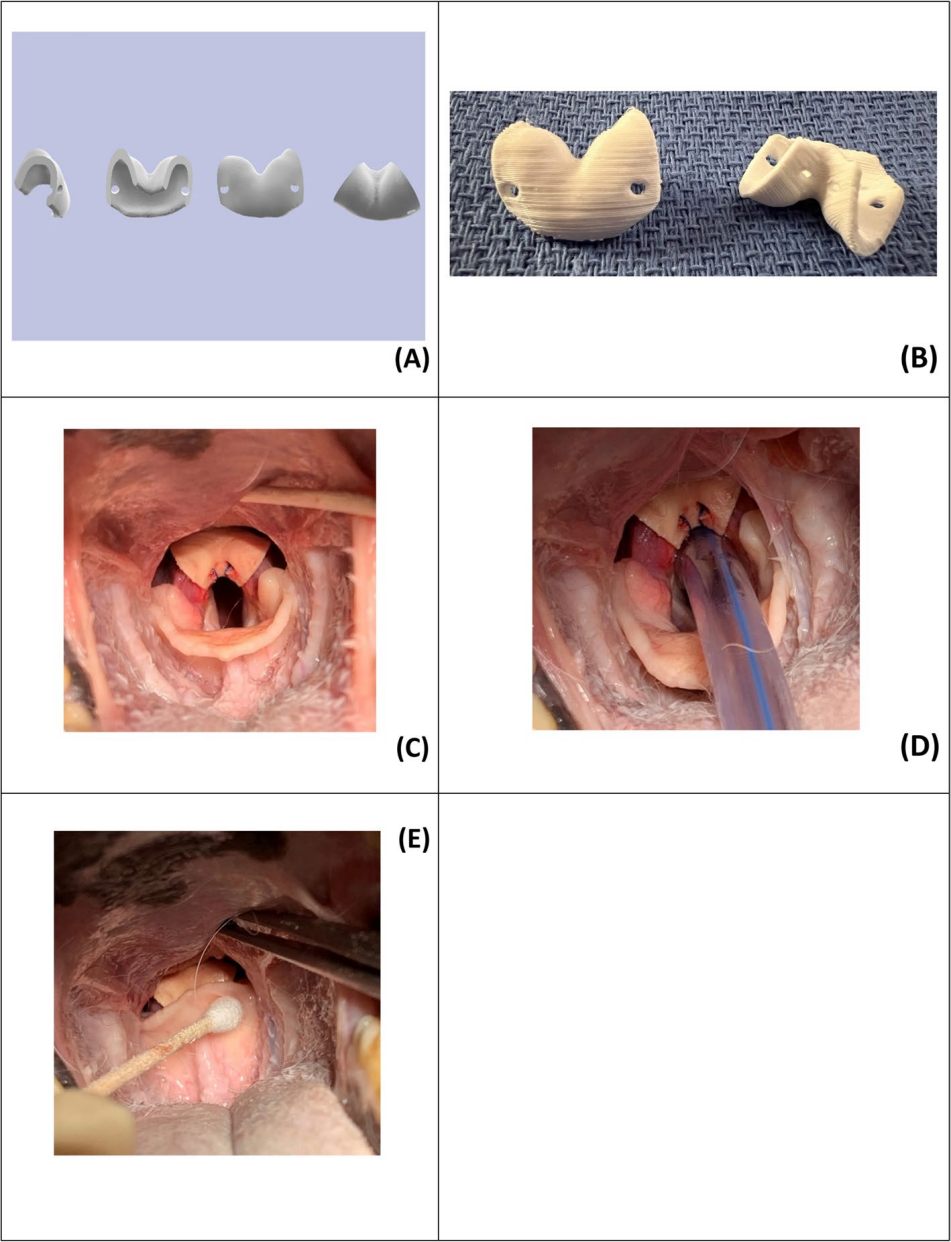
Computer generated renderings of the bilateral arytenoid abductor using Autodesk Meshmixer (**A**). Dorsal and ventral views of 3D-printed arytenoid abductor (**B**). Arytenoid abductor secured in situ (**C**), with an endotracheal tube in place (**D**) and with the epiglottis closed (**E**)

The bilateral arytenoid abductor device was created on Autodesk Meshmixer (Fig. 2A). The shape of the abductor was based off 3D reconstructed CT images of a larynx from an unaffected Labrador Retriever with an appropriately sized endotracheal tube in place. The width of the rima glottidis opening and the width of the epiglottis were measured in each cadaver at the level of the cuneiform processes with calipers. The shape of the abductor was designed to snuggly fit over top of the corniculate processes (Fig. 2). Six different sizes were created by changing the scale of the overall device size by 10% and the rima glottidis width by 15% across the different models (Autodesk Fusion 360). This opening width of the devices ranged from 0.6–1.6 cm to accommodate different laryngeal sizes. These models were then 3D printed in polylactic acid. Each abductor was placed along the top of corniculate processes by one investigator (KEM) and the rima glottidis width was measured (cm) at the level of the cuneiform tubercles with calipers. The size of the bilateral arytenoid abductor chosen for testing of a given cadaver larynx corresponded to the device size that increased the rima glottidis between 1.4—2 × the width measured in the control larynx. The resultant rima glottidis opening width had to be smaller than the measured width of the epiglottis at the level of the cuneiform processes to ensure appropriate laryngeal coverage with the epiglottis closed. Once the appropriately sized bilateral arytenoid abductor device was chosen it was then secured to the corniculate processes by passing two ligatures of 3–0 polydioxanone from caudal to cranial through small sutures holes present in the abductor (Fig. 2C). Each larynx was then mounted in the testing chamber and the airway pressure was measured with the epiglottis open and closed.

## Results

Mean ± SD width of the rima glottidis opening was 0.6 ± 0.3 cm in control specimens. Mean ± SD width of the epiglottis was 2.2 ± 0.4 cm in control specimens. After placement of the arytenoid abductor, mean width of the rima glottidis was 1.0 ± 0.4 cm.

With the epiglottis open, median laryngeal airway resistance in all specimens with a bilateral arytenoid abductor was significantly decreased at airflows of 15 L/min (0.0 cmH2O/L/sec), 30 L/min (0.2 cmH2O/L/sec), and 45 L/min (0.2 cmH2O/L/sec) compared to the control 15 L/min (0.4cmH2O/L/sec; *P* = 0.04), 30 L/min (0.9 cmH2O/L/sec; *P* = 0.04), and 45 L/min (1.2 cmH2O/L/sec; *P* = 0.04). At the highest airflow of 60 L/min, only 5 laryngeal specimens completed the testing because the smallest larynx collapsed secondary to high airway pressure during the control run. While there was a decrease in laryngeal airway resistance between the remaining 5 abducted larynges (0.4 cmH2O/L/sec) compared to controls (1.9 cmH20/L/sec), this decrease was not significant (*P* = 0.06) (Table 1).

**Table 1:** Laryngeal Airway Resistance (LAR = ΔP/V) with Epiglottis Open

**Flow** (L/min)	**Count**	**Median** (cmH20/L/min)	**25**^**th**^** percentile** (cmH20/L/min)	**75**^**th**^** percentile** (cmH20/L/min)	***P*-values**
15 control	6	0.4	0.2	1.2	0.04
15 abducted	6	0.0	0.0	0.2	
30 control	6	0.9	0.4	1.8	0.04
30 abducted	6	0.2	0.1	0.4	
45 control	6	1.2	0.5	2.2	0.04
45 abducted	6	0.2	0.1	0.6	
60 control	5	1.9	0.5	2.7	0.06
60 abducted	5	0.4	0.1	1.0	

Median ± 25^th^ and 75^th^ percentile laryngeal resistance at all airflows in control larynges and post placement of the bilateral arytenoid abductor with the epiglottis open. The number of larynges that were tested at each flow rate are included. At high flow rates during control testing, the smallest larynx collapsed

When the epiglottis was closed, there was no significant difference in laryngeal airway resistance between the control (18.8cmH_2_O/L/sec) and the abducted larynges (18.1cmH_2_O/L/sec; *P* = 0.83) (Table 2).

**Table 2:** Laryngeal Airway Resistance with Epiglottis Closed

**Flow** (L/min)	**Count**	**Median** (cmH20/L/min)	**25**^**th**^** percentile** (cmH20/L/min)	**75**^**th**^** percentile** (cmH20/L/min)	***P*-value**
10 control	6	18.1	13.5	67.8	0.83
10 abducted	6	18.8	12.9	48.7	

Median ± 25^th^ and 75^th^ percentile laryngeal resistance at the 10 L/min airflow in control larynges and post placement of the bilateral arytenoid abductor with the epiglottis closed. Six larynges were evaluated

## Discussion

We accepted our primary hypothesis that use of a bilateral arytenoid abductor would decrease laryngeal airway resistance with the epiglottis open. We also accepted our secondary hypothesis that there would be no difference in laryngeal airway resistance between controls and abducted larynges with the epiglottis closed.

Placement of a bilateral arytenoid abductor significantly reduced laryngeal airway resistance in canine cadaveric larynges with an open epiglottis at all airflows except the highest flow rate of 60 L/min. The failure to reach statistical significance at this flow rate could be due to loss of measurements secondary to collapse of the larynx with the smallest rima glottidis opening during control testing. The other 5 larynges showed a decreased laryngeal resistance at the 60L/min flow rate compared to control.

Cadaveric laryngeal specimens lack muscle tone and therefore the arytenoid cartilages are adducted and in the same paramedian position that would be observed in dogs with bilateral laryngeal paralysis. Previous studies evaluating the effectiveness of different surgical procedures for treatment of laryngeal paralysis have frequently used cadaveric canine larynges, validating the use of this method [4, 6, 20–22]. In this present study, airflows chosen for testing were similar to those reported in normal dogs [11, 23, 24], those affected by laryngeal paralysis [24], and similar to flows used in previous studies [4, 6]. Airflows > 60 L/min were not evaluated as previous investigators experienced laryngeal collapse during testing at airflows ≥ 90 L/min. [21].

Poiseuille’s law of resistance states that *R* = 8ηl/πr [4] where R is the resistance, n is gas viscosity, l is the length of the passageway, and r is the radius. This equation reveals that small changes of the radius of the airway can have large effect on the airway resistance. The significant reduction in laryngeal airway resistance experienced following placement of a bilateral arytenoid abductor compared to the controls in this study is explained by the increase in the rima glottidis opening width and prevention of paradoxical collapse of the rima glottidis during periods of high airflow. Because the rima glottidis is the most narrow portion of the airway, this increase in rima glottidis width increases the radius portion of Poisueille’s equation, resulting in a decreased resistance. The bilateral arytenoid abductor also allows both arytenoid cartilages to be abducted from the rima glottidis allowing for improved laminar flow, eliminating any turbulence that may disrupt airflow when there is one arytenoid cartilage remaining paralyzed and collapsed into the airway opening as would be seen following a unilateral arytenoid lateralization.

Previous studies have reported aspiration pneumonia as the most common complication after unilateral arytenoid lateralization, [13, 23] with one previous study reporting aspiration pneumonia occurring in 18.6%, 27.2%, and 31.8% of dogs 1-, 3- and 4 years following left unilateral arytenoid lateralization [14]. In our study, laryngeal resistance was not significantly different from the control after device placement with the epiglottis closed, which was a function of the positioning of the arytenoids following surgical intervention. Significant asymmetric, lateral abduction of one arytenoid cartilage as seen with the unilateral arytenoid lateralization promotes loss of the epiglottic seal as the arytenoid is pulled away from the cranial edge of the epiglottis [4, 19, 20]. With the bilateral arytenoid abductor, both arytenoid cartilages are symmetrically abducted a lesser magnitude allowing for maintenance of the contact between the epiglottic edge and the arytenoids, maintaining the seal that protects from aspiration. The size of the abductor that can be used would therefore be limited by the size of the epiglottis which can be measured preoperatively when a patient is intubated to aid with choosing a device size. An abductor that opened the arytenoid cartilages beyond edges of the epiglottis would likely have an associated increased risk for aspiration pneumonia. Excessive retraction of the arytenoid cartilages beyond the epiglottic-glottic seal may explain why patients who receive bilateral arytenoid lateralization have previously been reported to have a significantly increased risk for postoperative pneumonia [13].

The unilateral arytenoid lateralization is a technically demanding procedure with soft tissue dissection often necessitating postoperative opioid pain control. In addition, complications such as seroma formation have been reported to occur at the surgery site in up to 10% of patients [12]. Elimination of surgical dissection for treatment of laryngeal paralysis may decrease the need for postoperative opioids which have been associated with increased risk of postoperative aspiration pneumonia [14]. Placement of the bilateral arytenoid abductor per os would also eliminate the need for surgical preparation therefore decreasing anesthesia time. There are however, potential negative effects from placing an abductor at the level of the larynx; complications such as chronic irritation or chondritis of the arytenoid cartilages. Further testing is required to determine the material that would be best suited to provide enough rigidity to keep the arytenoids abducted without causing significant irritation. The abductors also have the potential to become dislodged from the arytenoids. The authors felt that because this abductor would be used to treat a disease occurring secondary to paralysis of the larynx that there would be little motion of the laryngeal cartilages to disrupt the abductor. Suture breakage or disruption from material passing by the abductor during swallowing could lead to dislodgement of the abductor into the airway or rotation of the abductor along the cartilages. In vivo testing will be required to determine how patients would tolerate this device prior to recommendation for clinical use.

The present study has several limitations, including the small sample size and use of cadaveric canine larynges. While in most testing scenarios we were able to achieve a statistical significance, at the highest air flow rate with the epiglottis open we could not achieve statistical significance with the collapse of one of the larynges during control testing; drawing experimental conclusions from a small testing population can result in type II statistical error. Studies using cadaver larynges are limited due to the loss of supporting perilaryngeal tissues, loss of muscular tone, and lack of response to tissue balance [21]. In this study, each larynx served as its own control because each specimen underwent testing in all conditions allowing for direct comparison between control and surgically treated larynges. Testing in canine cadavers does not allow for evaluating the larynx throughout a normal respiratory cycle but airflows used in this study were similar to those previously reported in both normal dogs [11, 23, 24] and those affected by laryngeal paralysis [24].

Another limitation of this study was the use of positive pressure airflow from the cranial aspect of the larynx rather than negative suction airflow from the caudal aspect of the larynx as would be experienced in vivo. Airway pressures measured in this study were similar to those previously reported in a study that used a vacuum at the caudal aspect of the larynx, [20] suggesting that the direction of airflow does not play a significant role in airway resistance calculation.

## Conclusions

We concluded that a bilateral arytenoid abductor successfully lowers laryngeal airway resistance when the epiglottis is open while also maintaining closed epiglottis laryngeal resistance. Maintenance of resistance when the epiglottis is closed is suggestive of maintenance of the epiglottic-glottic seal. In a clinical setting, the arytenoid abductor may be able to reduce laryngeal airway resistance without the increased risk for postoperative pneumonia. A trial using the bilateral arytenoid abductor in dogs affected with laryngeal paralysis is needed to determine the ideal material that will provide appropriate rigidity without causing laryngeal irritation and to evaluate if this device will be tolerated in vivo.

## Data Availability

Raw data were generated with RIT Bioprint at the Rochester Institute of Technology. Derived data supporting the findings of this study are available from the corresponding author [KM] upon request.
